# A Brief Report on the Factor Structure of the Cognitive Measures in the HRS/AHEAD Studies

**DOI:** 10.1155/2014/798514

**Published:** 2014-05-28

**Authors:** A. Nayena Blankson, John J. McArdle

**Affiliations:** ^1^Department of Psychology, Spelman College, 350 Spelman Lane S. W., Box 259, Atlanta, GA 30314, USA; ^2^Department of Psychology, University of Southern California, 711 Seeley G. Mudd Building, Los Angeles, CA 90089, USA

## Abstract

Using cognitive data from the Health and Retirement Study and Asset Health Dynamics Among the Oldest Old studies that were collected between 1992 and 2004, McArdle and colleagues (2007) found that a two-factor model (episodic memory and mental status) fit better than a one-factor model. The question that was addressed in the present study was whether these results would replicate in newer cohorts of data, collected between 2006 and 2010. We also tested age, education, and gender as predictors of the identified factors. Results confirm that a two-factor structure fits better than the single-factor model in the newer cohorts. Differential predictors were also observed.

## 1. Introduction

The measurement of intelligence is an important area of research in psychology and, with the increased longevity among Americans, cognitive aging has become a pertinent issue. Studies of cognition in older individuals can lead to increased understanding of the role of cognitive decline in everyday functioning among an increasingly aging population. However, in most investigations, the measurement of cognitive abilities is often treated as a unitary construct—usually referred to as general intelligence or general cognitive functioning. There is now considerable evidence to challenge that simplified view: a one-factor theory of intelligence does not explain many important observed relationships between intelligence and other variables (see [1], for a review). This finding has been observed in many previous investigations. In particular, recent research by McArdle et al. [2] examined the factor structure underlying the cognitive measures in the Health and Retirement Study (HRS) and Asset Health Dynamics Among the Oldest Old (AHEAD) studies. Results provided support for a two-factor model, one factor representing mental status and the second factor representing episodic memory. The purpose of the present study was to determine whether results obtained by McArdle et al. [2] would replicate to a new cohort of data. Additionally, we examined several demographic variables as predictors of the identified factors.

## 2. Literature Review

For over 100 years, researchers have investigated the measurement of cognitive abilities, from populations ranging from infancy to older ages. With increased longevity of individuals, strengthening our ability to measure cognitive skills of older individuals will help to facilitate research on predictors and outcomes associated with cognitive aging at older ages. The HRS and AHEAD studies have been one of the most widely used data sets in addressing research questions associated with cognition in the older ages. For example, Suthers et al. [3] investigated the link between life expectancy and cognitive impairment. Moody-Ayers et al. [4] examined the effect of cognitive functioning on functional decline. Not often considered in past research was the factor structure underlying the cognitive measures. Yet, more recent research on intelligence and cognition has shown that there is added value in determining whether we are measuring one and only one factor or multiple factors. Such arguments have at their roots the work of Spearman [5], which suggested that all human abilities can primarily be classified under one factor, and the work of Cattell [6] and Horn [1], which suggested that multiple intelligences better represent human cognition. The accumulated evidence to date supports a multiple-factor theory. Thus, it is important to consider whether a multiple-factor theory will also obtain in measures of cognition in the older ages.

Using cognitive data from the HRS and AHEAD studies, McArdle et al. [2] found that a two-factor model (episodic memory and mental status) fit better than a one-factor model. Since the publication of that paper, three new cohorts have been added to the data. Thus, a question remains as to whether conclusions reached by McArdle et al. that were based on data collected between 1992 and 2004 are generalizable to the newer cognitive data set, collected between 2006 and 2010. According to Lykken [7], constructive replication is when original hypotheses are tested with new methodology. Constructive replication helps to provide stronger support for a theory by demonstrating that conclusions are not restricted to a specific method. The present analyses serve as a replication of the research conducted by McArdle et al. That is, in the present investigation we test hypotheses tested by McArdle et al. [2] using more recent data. Specifically, we tested (1) whether a 2-factor model fits better than a 1-factor model and (2) age, education, and gender as the predictors of the identified factors. These variables have been extensively studied in past research as predictors of cognition. Therefore, inclusion of these same predictors in the present analyses is of scientific import.

The Health and Retirement Study (HRS) and Asset Health Dynamics Among the Oldest Old (AHEAD) studies began in 1992 and 1993, respectively, and in 1998 were combined into one study that attempts to be nationally representative of Americans over 50 years of age. The studies use a panel design in which the same respondents are interviewed every two years, and new respondents are added to the sample every six years to replenish the sample to adjust for aging and attrition (see [8, 9]; http://hrsonline.isr.umich.edu/).

The cognitive performance tests in the HRS/AHEAD studies measuring episodic memory and mental status were used in the present study. Specifically, the immediate and delayed free recall tasks have been found to measure an episodic memory factor while a mental status factor is comprised of the serial 7s, counting backward from 20, naming, and dates tasks. For the immediate and delayed recall tasks, respondents are asked to recall a list of nouns read by an interviewer immediately and after a 5-minute delay [10]. Random assignment was used within time points to assign the list of words for recall. For the serial 7s task, participants are asked to subtract 7 from 100 across 5 trials. On the counting backward task, individuals count backward from 20 for 10 continuous numbers. For the names task, respondents state the US president and vice president by last name and name two objects (scissors and cactus). Finally, for the dates task, respondents provide the current date (month, day, year, and day of week). These tests were adapted from the Telephone Interview of Cognitive Status (TICS; [11]), which itself was adapted for telephone administration from the Mini-Mental State Exam (MMSE; [12]). Though the MMSE is one of the most widely used quick screen measures of cognition for diagnosing cognitive impairment, the HRS cognitive measures can be argued to be among the most widely used measures for assessing cognition in older individuals for research purposes, given the sample size of the HRS data. Thus, the HRS data provide a rich source of information on cognitive aging in the US. The data have been used extensively in the past, and it is anticipated that the data will continue to be used to study cognitive declines and their correlates among older individuals in the US. The present research will therefore inform researchers on the best practices for use of these data in addressing questions regarding cognitive functioning among older Americans.

## 3. Method

### 3.1. Participants

The HRS is a nationally representative longitudinal study sponsored by the National Institute on Aging and conducted by the University of Michigan. The HRS researchers targeted community-dwelling adults in the contiguous United States who were 51 to 61 years old in 1992, when the baseline interview was conducted. Blacks, Hispanics, and Florida residents were oversampled (for details, see [8]). In 1993 and 1995, the AHEAD study was conducted among a national sample of adults aged 70 or older. In 1998, the HRS and AHEAD studies merged, both assuming the name HRS, and two new cohorts were added to the HRS sampling frame. New cohorts are added every two years.

For the present study, we used data collected from Waves 8 (2006) through 10 (2010). More specifically, as part of the data cleaning process, previous researchers (i.e., [13]) imputed missing cognitive data for participants using a multivariate, regression-based procedure using Imputation and Variance Estimation (IVEware) software. Our initial sample (*N* = 361) is compromised of those participants from the 2006 to 2010 waves who had data (imputed or self-respondent) available on the cognitive variables of interest. We then eliminated any person who had a sampling weight of zero (*n* = 133) or missing data on the cognitive variables at the first occasion of testing (*n* = 11), resulting in the subsample of 217 respondents described in Table 1. The demographic variables presented in this table include (a) chronological age at baseline testing, (b) years of formal education, and (c) gender.

### 3.2. Measures

The cognitive performance tests in the HRS/AHEAD included immediate and delayed free recall, serial 7s, counting backwards from 20, naming the US president and vice president by last name, naming two objects (scissors and cactus), and providing the date (month, day, year, and day of week). For the recall tasks, participants recalled a list of 10 words. Individuals received a score of 1 point for each word recalled correctly. Regarding the serial 7s task, the respondents were asked to start from the number 100 and subtract 7 continuously, for up to 5 trials. Participants received 1 point for each correct subtraction among the 5 trials, with each subtraction scored independently. For the counting backward task, participants received two trials, in which they were required to count backward from 20 for 10 continuous numbers. If participants responded correctly on the first trial, they received 2 points. If participants were correct on the second try, they received 1 point. Individuals who failed on both opportunities received 0 points. For the names task, respondents had to name the current US president and vice president by last name and name two objects. Each of the 4 names was scored independently, with 1 point for correct responses and 0 points for incorrect responses, and a total score was computed across the 4. Finally, for the dates task, respondents had to provide the current date (month, day, year, and day of week). Similar to the scoring of the names task, each of the 4 aspects of the date received 1 point for a correct answer and 0 points for an incorrect answer, and a total score was computed across the 4 dates.

To provide comparability across all scales and to simplify measurement for further statistical analysis, we scaled each variable into percent correct scores (i.e., based on division by the maximum score and multiplication by 100). The serial 7s, backward counting, dates, and names tasks were all skewed and therefore treated as categorical variables in the analyses.

### 3.3. Data Analyses

Substantive analyses included confirmatory factor analyses conducted to test the 1- and the 2-factor models and latent factor path modeling, to examine age, education, and gender as predictors of cognition. For the 2-factor model, the first factor was marked by two continuous variables (immediate recall and delayed recall), while the second factor was marked by four categorical variables (serial 7s, backward counting, dates, and names). Weighted least squares with mean and variance adjusted (WLSMV) estimation was used to account for the skewed categorical variables. Use of WLSMV estimation has been found to perform better than maximum likelihood estimation when data are categorical [14]. Delta parameterization was employed so that scale factors could be modeled for the categorical variables. Factors were identified by fixing the loadings for immediate recall and serial 7s at unity. Analyses were conducted using Mplus 7.0 [15]. In all analyses, goodness-of-fit indices were used to make decisions about the accuracy of the models. More specifically, overall *χ* ^2^ is presented and we also rely on the root-mean square error of approximation (RMSEA; [16, 17]) and comparative fit index (CFI; [18]) for the assessment of good fit. As a rule of thumb, RMSEA values smaller than 0.10 [16] and CFI values above 0.95 [19] were considered favorable although CFI values above 0.90 are tenable [18] and are still a widely used cut-off [20].

Once we establish the underlying factor structure among the cognitive variables, then we can begin to address questions regarding predictors of cognition in older ages. To address this question, the latent factors were regressed on age, education, and gender.

## 4. Results

Summary statistics on all cognitive data are presented in Table 2 and in Table 3 are the correlations among the cognitive variables. This information is based on the cognitive variables at the first time of testing for all participants. Immediate recall (IR) had an average near 50% but the delayed recall (DR) scale was somewhat harder. Backward counting (BC), dates (DA), and names (NA) had over 90% correct response rate and were negatively skewed.

Next, confirmatory factor modeling was applied to test the 1-factor versus 2-factor hypothesis. The 2-factor model fit better. The fit of the 1-factor model was chi-square = 53 (df = 9); CFI = 0.79; RMSEA = 0.15 (95% CI = 0.19; 0.19). The fit of the 2-factor model was chi-square = 20 (df = 8); CFI = 0.94; RMSEA = 0.08 (95% CI = 0.04; 0.13). Standardized factor loadings for the 1- and 2-factor models are displayed in Table 4.

Results of the latent factor path model predicting the two factors are shown in Table 5. Results revealed significant age and education effects for episodic memory. However, gender differences were observed only for episodic memory but not mental status.

## 5. Discussion

Recent research in cognitive aging has focused on determining the factor structure underlying cognitive tests. More specifically, research by McArdle et al. [2] suggested that a 2-factor structure was a better fit to the cognitive tests in the HRS/AHEAD study than a 1-factor structure. The present study served as a replication of the McArdle et al. analyses by testing the factor structure in data collected between 2006 and 2010. Additionally, we tested age, education, and gender as predictors of the obtained factors. Results provided support for the 2-factor model, consistent with McArdle et al. [2].

The primary question that was addressed in the present investigation was whether the 2-factor structure would hold up in the new cohorts, and results indicate that this is indeed the case. Moreover, examination of the predictors of the factors indicates that while age and education predict both factors, gender predicted only the episodic memory factor. Younger individuals were found to perform better than the older participants, as did those with higher levels of education. Thus, in using these cognitive tests for research or clinical purposes, it is important to take these variables into consideration. Females were also found to perform better on the test of episodic memory than were males, but this same gender difference was not observed for mental status. The differential effects of gender on the two factors highlight the importance of considering the cognitive tests using a multiple-factor framework rather than a single-factor framework in future research.

Rarely are results replicated and published in research. Often, the argument is made that the replicated results do not add new information to the literature and, therefore, most replication studies go unpublished. Yet, it is through replication that we can enhance our understanding regarding various psychological phenomena, particularly because most of the research conducted in the fields of psychology and gerontology is correlational in nature. Thus, the primary aim of the present research presents an inherent strength of the study. Despite this strength, however, there is the limitation of the small sample size of the current data, precluding such tests such as invariance of measurement across groups. Nevertheless, the results indicate that researchers should use a 2-factor representation of the cognitive data rather than the 1-factor representation in future research. Moreover, because the tests that were examined in the present research are an adaptation of the Mini-Mental State Exam, these results also suggest that it may be of benefit to consider the 2 components as separate aspects of cognitive functioning in clinical screening of patients.

## Figures and Tables

**Table 1:** Sample demographics at initial testing.

	Age in years	Years of education	Female (%)
M	66.63	13.12	47
SD	9.25	2.88	0.50
Minimum	52.00	0.00	0 = male
Maximum	93.00	17.00	1 = female

**Table 2:** Descriptive statistics for cognitive measures at initial testing.

Statistic	IR	DR	S7	BC	DA	NA
M	53.96	42.49	70.69	96.77	94.35	91.71
SD	16.58	19.56	31.39	17.71	11.78	14.84
Min	10	0	0	0	50	25
Max	100	100	100	100	100	100
Skewness	0.27	0.10	−0.74	−5.33	−1.97	−1.75
Kurtosis	0.22	0.14	−0.69	27.67	3.14	2.60

Note: IR: immediate recall; DR: delayed recall; S7: serial 7s; BC: backward counting; DA: dates; NA: names.

**Table 3:** Correlations among cognitive measures.

Variable	1	2	3	4	5
(1) Immediate recall	—				
(2) Delayed recall	0.76**	—			
(3) Serial 7s	0.27**	0.27**	—		
(4) Backward counting	0.08	0.09	0.06	—	
(5) Dates	0.26**	0.33**	0.26**	0.13*	—
(6) Names	0.20**	0.27**	0.36**	0.21**	0.33**

**P < 0.01.

*P < 0.05.

**Table 4:** Standardized loadings from confirmatory factor analyses.

	Memory		Mental status^a^			
	IR	DR	S7	BC	DA	NA
One-factor model						
Loading	0.78	0.84	0.49	0.20	0.72	0.62
Uniqueness	0.40	0.30	0.76	0.96	0.49	0.62
Two-factor model						
Loading	0.90	0.90	0.57	0.28	0.86	0.72
Uniqueness	0.19	0.19	0.67	0.91	0.27	0.48

^a^Uniqueness parameters estimated from thresholds.

**Table 5:** Parameter estimates for prediction of two factors (standardized estimates in parentheses).

Factor predictor	Episodic memory	Mental status
Age	−0.44 (−0.24)**	−0.02 (−0.22)*
Education	1.96 (0.34)**	0.11 (0.47)**
Female	8.58 (0.28)**	−0.19 (−0.15)

**P < 0.01.

*P < 0.05.

## References

[1] Horn JL, Blankson AN, Flanagan DP, Harrison PL (2012). Foundations for better understanding of cognitive abilities. *Contemporary Intellectual Assessment: Theories, Tests, and Issues*.

[2] McArdle JJ, Fisher GG, Kadlec KM (2007). Latent variable analyses of age trends of cognition in the health and retirement Study, 1992–2004. *Psychology and Aging*.

[3] Suthers K, Kim JK, Crimmins E (2003). Life expectancy with cognitive impairment in the older population of the United States. *Journals of Gerontology B: Psychological Sciences and Social Sciences*.

[4] Moody-Ayers SY, Mehta KM, Lindquist K, Sands L, Covinsky KE (2005). Black-white disparities in functional decline in older persons: the role of cognitive function. *Journals of Gerontology A: Biological Sciences and Medical Sciences*.

[5] Spearman C (1904). General intelligence: objectively determined and measured. *The American Journal of Psychology*.

[6] Cattell RB (1941). Some theoretical issues in adult intelligence testing. *Psychological Bulletin*.

[7] Lykken DT (1968). Statistical significance in psychological research. *Psychological Bulletin*.

[8] Heeringa SG, Connor JH (1996). *Technical Description of the Health and Retirement Study Sample Design*.

[9] Leacock C (2006). *Getting Started with the Health and Retirement Study, Version 1. 0*.

[10] Ofstedal MB, Fisher GG, Herzog AR (2005). Documentation of cognitive functioning measures in the Health and Retirement Study. *HRS/AHEAD Documentation Report*.

[11] Brandt J, Spencer M, Folstein M (1988). The telephone interview for cognitive status. *Neuropsychiatry, Neuropsychology and Behavioral Neurology*.

[12] Folstein MF, Folstein SE, McHugh PR (1975). Mini-Mental State: a practical method for grading the cognitive state of patients for the clinician. *Journal of Psychiatric Research*.

[13] Fisher GG, Hassan H, Rodgers WL, Weir DR (2012). *Health and Retirement Study Imputation of Cognitive Functioning Measures: 1992–2010 Early Release*.

[14] Beauducel A, Herzberg PY (2006). On the performance of maximum likelihood versus means and variance adjusted weighted least squares estimation in CFA. *Structural Equation Modeling*.

[15] Muthén LK, Muthén BO (1998–2012). *Mplus User's Guide*.

[16] Browne MA, Cudeck R, Bollen KA, Long JS (1993). Alternative ways of assessing model fit. *Testing Structural Equation Models*.

[17] Steiger JH, Lind JC Statistically-based tests for the number of common factors.

[18] Bentler PM (1990). Comparative fit indexes in structural models. *Psychological Bulletin*.

[19] Hu L-T, Bentler PM (1999). Cutoff criteria for fit indexes in covariance structure analysis: conventional criteria versus new alternatives. *Structural Equation Modeling*.

[20] Van Lieshout RJ, Cleverley K, Jenkins JM, Georgiades K (2011). Assessing the measurement invariance of the Center for Epidemiologic Studies Depression Scale across immigrant and non-immigrant women in the postpartum period. *Archives of Women’s Mental Health*.

